# Redefining the documentation of outdoor surface scatter scenes using geographic information systems

**DOI:** 10.1111/1556-4029.15698

**Published:** 2025-02-07

**Authors:** Katie Pottage, Peter Masters, Nicholas Márquez‐Grant

**Affiliations:** ^1^ Cranfield Forensic Institute Cranfield University Cranfield UK

**Keywords:** dGPS, forensic archaeology, GIS, scatter scene, scene documentation, spatial analysis, surface deposition, total station

## Abstract

The field of forensic archaeology has been primarily associated with the search, location, and excavation of clandestine graves, and thus, other deposition types have been commonly neglected in research. Current literature typically addresses the use of traditional methods implemented for the excavation and recovery of human remains from clandestine graves but fails to provide the same for surface scatter scenes. This study aimed to explore the documentation of such scenes through the integration of traditional archaeological techniques, geophysical surveying techniques, and GIS. A mixed method study was created and utilized in three different simulated scatter scenes, allowing the qualitative and quantitative scope of GIS to be examined and assessed. The techniques were utilized successively and iterated until all simulated scenes had been documented. Within this study, terrain was the independent variable—this was nonrandomized and chosen to best suit sites where scatter scenes are most prevalent. Results demonstrated GIS to be an effective method in the documentation of contextual data at a forensic surface scatter scene, providing both qualitative and quantitative data. Such findings aid in understanding the admissibility of each technique in court and its impact on a case when presented as evidence. This research revealed that further exploration of surveying techniques in sites other than clandestine graves is necessary for forensic archaeology practice.

**Highlights**

- This paper explored the use of GIS in surface scatter scenes.
- Three difference surface scenes with plastic skeletal elements were documented.
- The study compared results from trilateration, total station, and differential GPS.
- This research revealed that GIS serves as a useful tool in the documentation of scatter scenes.
- The terrain environment is not critical to the usefulness, accuracy, or efficiency of GIS.

## 1 INTRODUCTION

Rural environments (woodlands, hedge clearings, and open fields) are common deposition sites for human cadavers in a number of forensic cases, often as a result of homicide or suicide [1, 2, 3]. Being a less‐densely populated environment, rural terrains are typically perceived by perpetrators to be an appropriate disposal site for cadavers—particularly in cases of homicide where mutilation or dismemberment has occurred [4, 5, 6]. In addition, the number of suicides is significantly more prevalent in rural environments [7, 8]. Statistics reveal that between 2010 and 2018 in the United Kingdom, rural suicide rates increased from 8.8 deaths (per 100,000 people) to 10.9 compared to urban environments, which saw suicide rates increase from 9.3 deaths (per 100,000 people) to 10.3 [9].

Human skeletonized remains that are found disarticulated (i.e., not in anatomical connection or joined by soft tissue) and spread over an area of land are typically referred to as a “scatter scene” or “scatter surface scene.” While scatter scenes themselves have no official definition, there are two types that are commonly observed: (1) remains that are exposed on the ground surface, and (2) remains concealed by leaf litter or other material [10]. In cases of skeletonized remains in the United Kingdom, it is the responsibility of the crime scene investigators or scenes of crime officers—alongside a forensic archaeologist or anthropologist if possible—to document the scene and its context using a multidisciplinary approach, typically through the application of various scientific (including archaeological) techniques [11, 12]. At indoor scenes, the risk of contamination and evidence preservation is easier to maintain, enabling protocols to render adequate scene documentation solely through qualitative techniques [11, 12, 13]. This is more difficult in outdoor scenes where there are a greater number of variables—including animal scavenging, weather, and root action, among others—and thus, the documentation guidelines for these scenes are neither robust nor exhaustive [12, 14, 15, 16].

Qualitative methods typically involve the collection and analysis of non‐numerical data to visualize the scene (photographs, scene sketch plans, and written documentation), whereas quantitative methods focus on the use of statistics, measurements, and observed correlations [17]. A Geographic Information System (GIS) is an example of software that can be utilized for both qualitative and quantitative purposes. It offers a “user‐defined complexity” for the capture, display, and analysis of geospatial data, allowing information to be refined and manipulated depending on the desired outcome [18, 19].

Originating in the early 1850s as paper mapping, GIS was initially implemented as a law enforcement tool before being applied to geographic profiling to determine probable areas of offenses [3, 20, 21, 22, 23]. Nevertheless, the utilization of GIS has still not reached its full potential in crime scene investigation. Based on publications such as Orengo (2007) [24] and Somma et al. (2018) [25], GIS can assist in the detection of clandestine burials. For example, through ordnance survey maps, Orengo (2007) [24] layered environmental features such as water bodies, road locations, and bore holes to produce a vector model, demonstrating the areas where clandestine burials were more likely to be found. Comparatively, Somma et al. (2018) [25] adopted a quantitative approach and implemented probability mapping through a red, amber, green (RAG) color‐coded system. While both publications proved GIS to be successful when applied either qualitatively or quantitatively to the detection of clandestine burials, De Leeuwe and Groen (2015) [26] highlight its contribution in identifying the position and displacement of the skeletal elements within scatter scenes. To put this into perspective, the work of De Leeuwe and Groen (2015) [26] depicts a qualitative approach to the use of GIS and provides the user with a visualization of the scene that can be used alongside photographs and scene sketches. However, the application of this software did not extend beyond this.

### 1.1 Aims and objectives

This paper aims to: (1) establish how the efficiency of GIS at surface scatter scenes alters with differing terrain, (2) ascertain the overall applicability and accuracy of GIS when applied to scatter scenes, and (3) promote the usefulness of GIS as a resource in this field. In order to address these aims, a number of objectives were set in this study: (1) map scatter scenes at three different locations, (2) document each scene using traditional mapping techniques, terrestrial surveying methods, and GIS, and (3) establish how the documentation of scatter scenes varies with differing terrain.

## 2 MATERIALS AND METHODS

Due to ethical reasons and in accordance with The Human Tissue Act 2004 for England and Wales [27], faux skeletal elements (plastic skeletons) were used to undertake the experimental phase of this project. The methodology follows three distinct phases: (i) Scene Construction, (ii) Mapping and Documentation, and (iii) Software Utilization.

### 2.1 Scene construction

This experiment was conducted at Cranfield University, Cranfield, MK43 0AL, in the United Kingdom. Chosen due to their isolated nature, three locations of the university's grounds were utilized. Of these locations, two were open fields, with the other being a woodland. The use of two open field environments allowed for one to act as a control scene.

At each location, scatter scenes were constructed within a 5 × 5 meter cordoned area. Plastic skeletal elements were dispersed within the 5 × 5 meter area with consideration of taphonomic factors such as animal scavenging, which are typically present at scatter scenes. Skeletal elements were occluded by vegetation and dispositioned by the sloping of the terrain, which would be typical due to gravity. This meant that the surface deposition sites were analogous to that of an actual scene, with the exception of the remains being positioned “anatomically.” The reasoning for such is considered within the discussion section of this paper.

The scatter scene sites were constructed in restricted airspace, with the experimental site being situated in close proximity to Cranfield Airport, thus limiting the mapping techniques utilized and preventing the use of drone mapping. Should the experimental site have been in a different location, a drone with Real Time Kinematic (RTK) GPS capabilities or Unmanned Aerial Vehicle (UAV) would have been integrated into the methodology.

### 2.2 Mapping and documentation

#### 2.2.1 Trilateration and scene sketch plan

The author (KP) first used the trilateration method to record the position of the skeletal elements. The number and location of each point taken were noted separately and can be observed in Table 1, alongside a skeletal inventory.

**TABLE 1:** An inventory showing the skeletal elements used, as well as the number of datapoints collected.

Skeletal element	Side		Number of datapoints obtained*	Location*
	Left	Right		
Skull	✓		1	Center point
Hyoid				
Vertebrae				
C1 (Atlas)	✓		1	Center point
C2 (Axis)	✓		1	Center point
C3–C7				
T1–T12				
L1–L5	✓^a^		1	Center point
Sacrum and Coccyx	✓		3	Proximal and distal aspects
Ribs				
1st				
2nd	✓	✓	3	Head, sternal end, and center point
3rd–10th	✓^b^	✓^c^	3	Head, sternal end, and center point
11th	✓		3	Head, sternal end, and center point
12th				
Sternum				
Clavicle	✓	✓	3	Medial, shaft, and lateral aspects
Scapula	✓	✓	3	Medial, lateral, and distal aspects
Os Coxae	✓	✓	4	Proximal, lateral, medial, and distal aspects
Long Bones				
Humerus	✓	✓	2	Proximal and distal ends
Radius	✓	✓	2	Proximal and distal ends
Ulna	✓	✓	2	Proximal and distal ends
Femur	✓	✓	2	Proximal and distal ends
Tibia	✓	✓	2	Proximal and distal ends
Fibula	✓	✓	2	Proximal and distal ends
Carpal Bones				
Scaphoid				
Lunate				
Capitate				
Triquetral				
Pisiform				
Trapezium				
Trapezoid				
Hamate				
Metacarpals				
1–5				
Hand Phalanges				
Proximal				
Intermediate				
Distal				
Tarsal Bones				
Calcaneous		✓	1	Center point
Talus		✓	1	Center point
Cuboid				
Navicular				
Medial Cuneiform				
Intermediate Cuneiform				
Lateral Cuneiform				
Metatarsals				
1–5		✓	1	Center point
Foot Phalanges				
Proximal				
Intermediate				
Distal				

^a^ With the exception of L5.

^b^ With the exception of the 9th rib.

^c^ With the exception of the 5th and 9th rib. The 6th rib was broken in two.

*Per side, or per skeletal element, where applicable.

A plotted scene sketch was then drawn up, utilizing the measurements obtained through the trilateration method. Such a sketch was mapped on graph paper using a ballpoint pen, where 3 centimeters was equivalent to 1 meter.

#### 2.2.2 Total station

A Leica TS16 was used to map the skeletal elements. The Leica TS16 was used to measure each skeletal element in accordance with Table 1, in addition to the four pegs that defined the scene perimeter. At each respective peg, the reflector was placed into the soil, recording the position of the remains within the 5 × 5 meter area.

#### 2.2.3 dGPS

A Trimble GeoXH GeoExplorer 3000 series Differential GPS (dGPS) was used to allow for sub‐meter accuracy. Such instrumentation was calibrated using the identical backsight and reference points as taken with the total station. Data points were collected in line with Table 1, as well as the four respective pegs placed in each corner of the scene.

### 2.3 Software utilization

The datapoints obtained from each instrumentation were converted into decimal degrees from The World Geodetic System 1984 (WGS84), before they were analyzed using GIS software, ArcGIS Pro. On the “Imagery” base layer, each set of data was collated and layered, allowing the scene to be plotted in a 2‐dimensional manner. Although data was subject to positional error during transformation between two coordinate systems, use of the correct transformation method mitigates against this.

To conduct quantitative analysis, accuracy buffers were established using the rate of error for each technique. Directed by the user manual guidelines [28, 29], respective accuracy buffers of 2 millimeters (0.002 meters) and 1 meter were applied to the datapoints, where 0.002 meters was applied to the total station data and 1 meter to the dGPS data. Quantitative analysis was conducted by assessing the spatial relationships between the corresponding skeletal elements at each scene. Here, feature analysis was conducted using the “Connect Origins to Destinations” tool, and with input of the necessary data requirements (e.g., input point layers with matching identification fields), the distance between each respective element as positioned by the total station and dGPS was determined. The settings were modified to ensure distance was calculated in meters, and such findings were presented in a standalone table.

**FIGURE 1:**
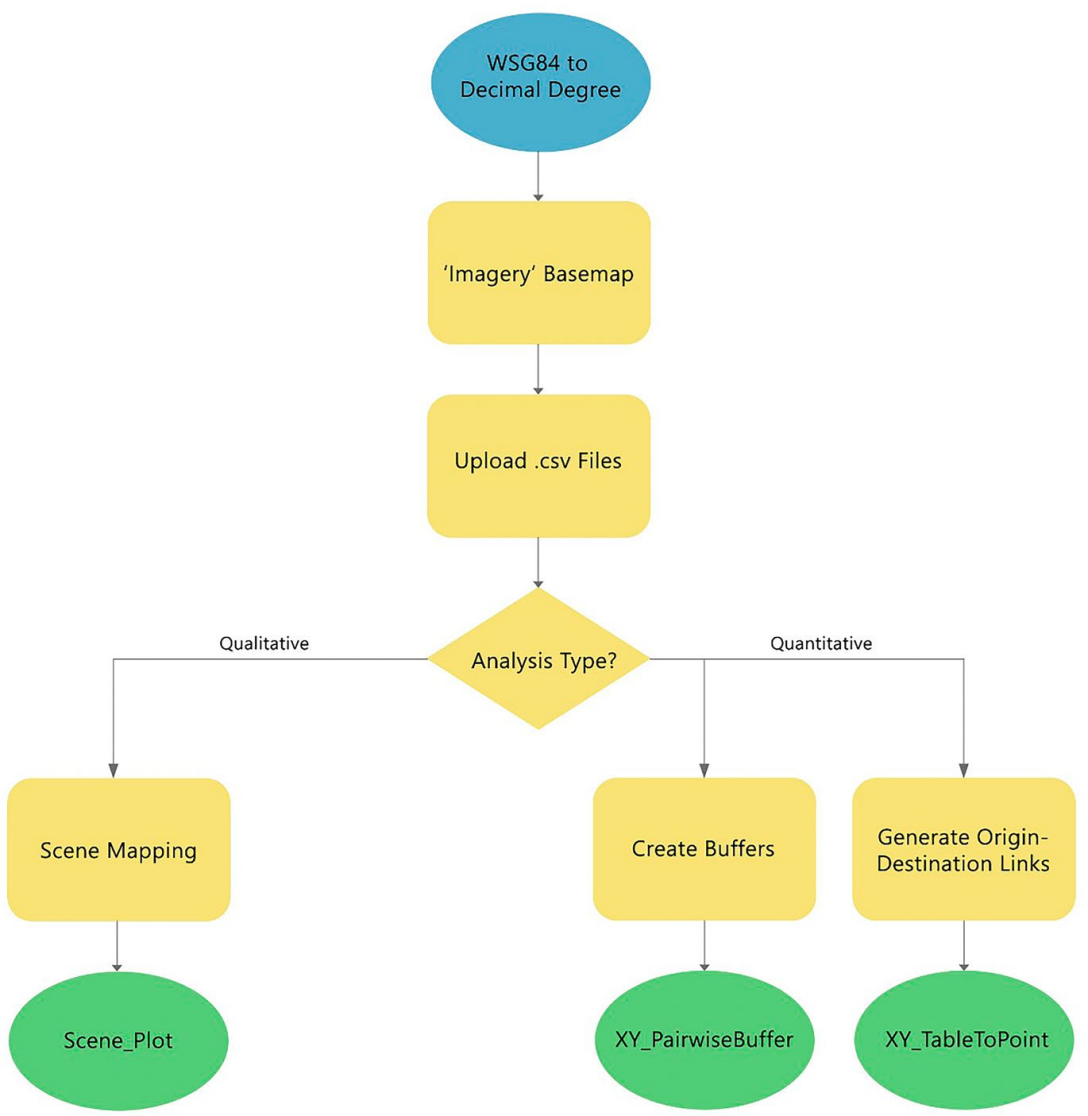
Workflow depicting the processes carried out in ArcGIS Pro.

Figure 1 depicts a workflow of the key processes required for software utilization.

## 3 RESULTS

Due to the number of points being obtained, the trilateration method, total station, and dGPS took approximately an hour and a half to complete individually. Where the trilateration method obtained a total of 112 points at each scene, the total station and dGPS, respectively, accumulated 118 datapoints (with the exception of one) due to the additional recording of the scene perimeter, reference point, and backsight. In wooded terrain, the dGPS seldom worked and was unable to obtain satisfactory satellite signal, resulting in only 8 datapoints being recorded.

### 3.1 Traditional mapping techniques

Completion of the plotted sketch was necessary and proved to be a useful representation of the scene. Although this technique was not impacted by terrain, the wooded environment did make it harder to complete the trilateration method—a necessity for the plotted sketch.

**FIGURE 2:**
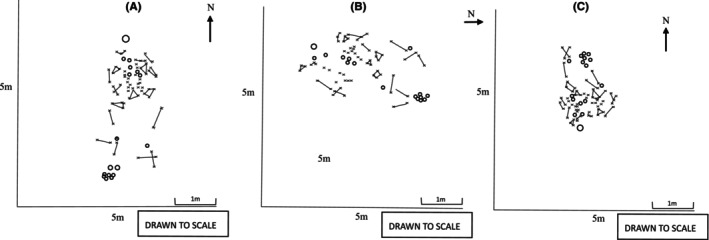
The three studied scenes were (A) is a traced representation of the plotted scene sketch plan from scene one, the control open field terrain; (B) the traced representation of the plotted scene sketch plan from scene two, the second open field terrain, and (C) the traced representation of the plotted scene sketch plan from scene three, the wooded terrain.

As demonstrated in Figures 1 and 2, the scale used to map each scatter did not have an impact on the positioning of each skeletal element, despite being amended to fit an A4 page. Features did not appear particularly congregated or distant, except for the ribs, which were notably crowded. As a result, only the proximal end of each rib was plotted.

It is important to note that Figure 2 includes traced versions of the original scene sketches drawn up by the first author (KP), as this allowed for a better visual representation within the results.

### 3.2 ArcGIS pro

#### 3.2.1 Qualitative

The data from each scatter scene simulation were observed as maps. The mapping of each respective scene revealed discrepancies with the geographic coordinates, as each set of datapoints was observed at different locations within Cranfield University.

Where the scatter within scene one appeared to be plotted in close proximity within the same field, such a trend was not continued at the other scenes. At scene two, the scatter was revealed to be in two separate locations: an open field and a public path when recorded by the dGPS and total station, respectively. Similarly, the scatter of the third scene was stationed in a woodland when plotted by the dGPS coordinates, while the total station placed the scatter in a field situated to the right of this woodland.

Moreover, each scatter of the dGPS resembled a loose anatomical position, whereas the datapoints of the total station are congregated in one area. Yet, from the data obtained by either instrumentation, it was not possible to determine which direction the skeletal elements were facing, nor was it exactly apparent where the limb bones (such as the ulna, radius, fibula, and metatarsals) were located. At both open scene environments, the datapoints also did not correspond to the location of the expected skeletal element. For example, at scene two, the points that were expected to be the right tibia and fibula were instead the distal end of the right radius and proximal end of the left ulna. Due to insufficient data, such a trend cannot be readily established at scene three.

#### 3.2.2 Quantitative

Accuracy buffers and spatial analysis allowed for interpersonal relationships between the datasets to be ascertained.

**FIGURE 3:**
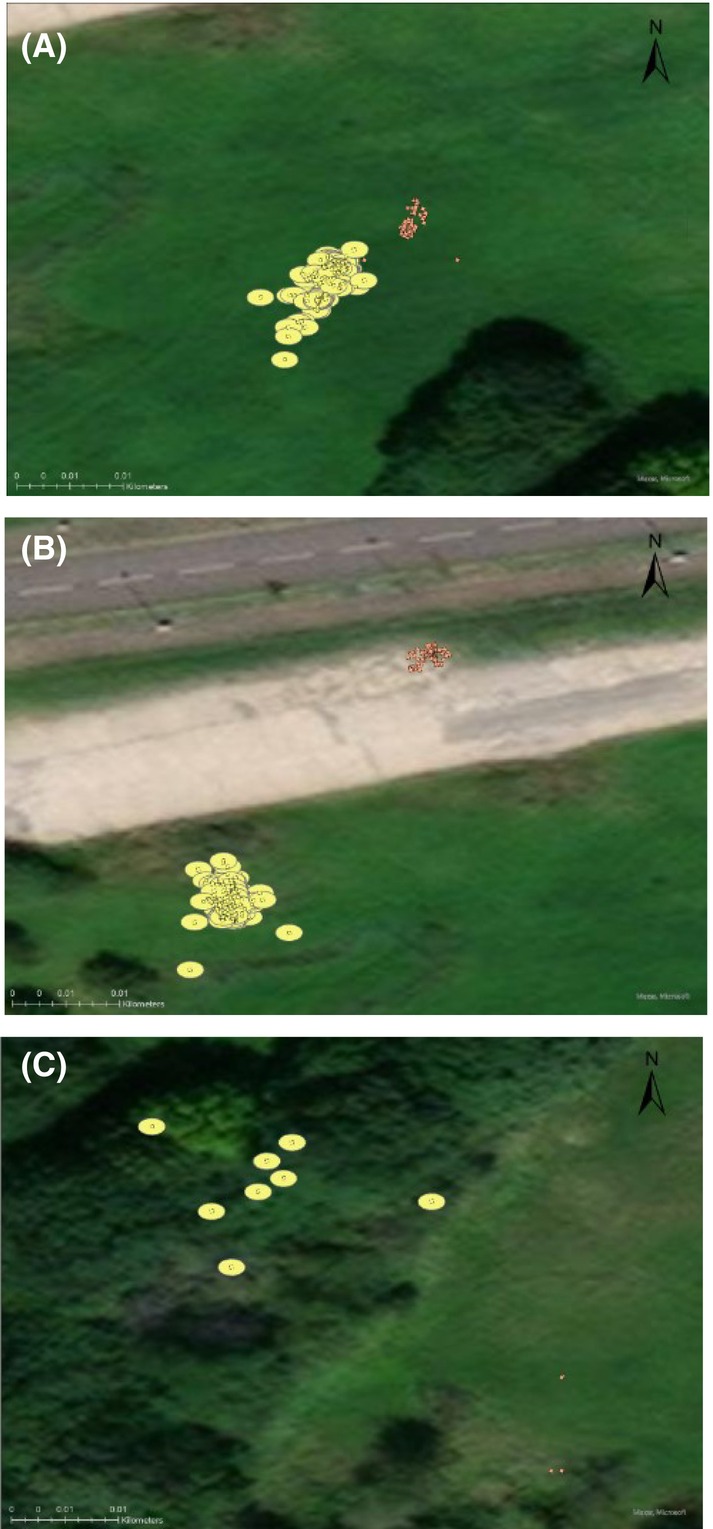
The three studied scenes with the plotted datapoints and their respective accuracy buffer as obtained by the total station and GPS. Each scene is shown at a scale of 1:300, where (A) is the open field control scene, (B) is the second open field scene, and (C) is the woodland scene (scene 3). The orange datapoints correspond to the total station data, while the GPS datapoints are yellow.

Figure 3 shows the dGPS to have the largest error level, while the total station has the smallest. Being 1 meter, the error level of the dGPS is 500 times greater than that of the total station, which at 0.002 meters was too minor to be plotted by ArcGIS Pro.

Furthermore, statistics were beneficial in calculating the spatial relations between the respective skeletal elements within the same scene (Table 2).

**TABLE 2:** The spatial relationship between all skeletal elements within each respective scene.

	Distance between skeletal elements plotted to 2dp (m)																					
Skeletal Element	Scene 1								Scene 2								Scene 3^a^					
	Left			Right					Left			Right					Left			Right		
**Skull**	11.96								29.67								49.52					
**Vertebrae**																						
C1 (Atlas)	10.02								29.60													
C2 (Axis)	10.02								29.49													
L1	9.97								29.61													
L2	9.85								29.54													
L3	9.72								29.34													
L4	9.38								29.34													
**Ribs**	**H**	**C**	**S**	**H**		**C**	**S**		**H**	**C**	**S**	**H**		**C**	**S**		**H**	**C**	**S**	**H**	**C**	**S**
2nd	9.56	9.32	9.46	9.29		9.34	9.22		29.68	29.70	29.66	29.60		29.86	29.67							
3rd	9.78	9.84	9.79	8.98		8.89	8.85		29.65	29.61	29.64	29.49		29.32	29.33							
4th	9.53	9.65	9.51	8.90		8.97	8.99		29.43	29.39	29.47	29.42		29.45	29.37							
5th	9.13	9.09	9.15						29.60	29.48	29.56											
6th	9.26	9.43	9.23	8.88	9.04		8.76	9.13	29.54	29.58	29.51	29.57	29.83		29.75	28.87						
7th	9.05	9.07	9.06	9.18		9.26	9.19		29.51	29.54	29.55	29.62		29.60	29.54							
8th	9.00	9.18	9.15	9.16		9.15	9.17		29.51	29.56	29.58	29.45		29.43	29.58							
10th	8.99	8.97	9.02	9.14		9.15	9.19		29.52	29.50	29.42	28.18		28.19	28.22							
11th	8.93	8.97	8.98						29.64	29.67	29.78											
**Clavicle**																						
Proximal	10.83			9.71					30.04			29.61										
Curve	10.84			9.65					30.02			29.61										
Distal	10.85			9.53					30.02			29.60										
**Scapula**																						
Medial aspect	11.02			9.61					29.43			29.63										
Lateral aspect	11.05			9.62					29.49			29.63										
Distal aspect	11.04			9.68					29.48			29.64										
**Os Coxae**																						
Proximal aspect	9.27			9.60					29.60			29.20					47.64					
Medial aspect	9.42			9.54					29.82			29.41										
Lateral aspect	9.48			9.55					29.54			29.60										
Distal aspect	9.47			9.56					29.48			29.41										
**Sacrum/coccyx**																						
Proximal aspect	9.83								29.31													
Proximal aspect	9.68								29.31													
Distal aspect	9.73								29.40													
**Long Bones**																						
**Humerus**																						
Proximal	9.55			9.73					29.01			29.40										
Distal	9.61			9.89					29.00			29.42										
**Radius**																						
Proximal	9.71			9.67					29.04			29.43										
Distal	9.65			9.71					29.18			29.55										
**Ulna**																						
Proximal	14.97			9.56					28.89			29.26										
Distal	9.59			9.78					29.05			29.37										
**Femur**																						
Proximal	9.37			9.37					29.87			29.21					47.64					
Distal	9.12			9.43					29.80			29.84					47.63					
**Tibia**																						
Proximal	9.24			9.39					29.80			29.61										
Distal	9.43			9.33					29.86			29.68										
**Fibula**																						
Proximal	9.23			9.14					29.53			29.73										
Distal	9.48			9.50					29.60			29.03										
**Patella**	9.42			8.95					29.63			29.30										
**Tarsal Bones**																						
Calcaneus				9.32								28.94										
Talus				9.25								29.30										
**Metatarsals**																						
1				9.18								29.08										
2				8.95								29.36										
3				9.08								29.34										
4				14.95								29.43										
5				9.30								29.35										

Abbreviations: C, rib center; H, head; S, sternal end.

^a^ Data obtained from the number of points that had been collected from both GPS and total station.

Table 2 shows the distance between the skeletal elements at each respective scene, as mapped by the total station and dGPS. The spatial distance between all skeletal elements remained relatively consistent at each scene, showed no significant variation, and was uninfluenced by terrain.

The two open field scenes exhibited vastly different results. At scene one, the average distance between each set of skeletal features was 9.37 meters. This distance increased slightly at the skull, atlas, axis, clavicle, and scapula, where these elements were observed to be between 10 and 12 meters apart. Two significant anomalies could be observed at the proximal left ulna and the fourth metatarsal. Both these skeletal elements exhibited respective distances of 14.97 meters and 14.95 meters—distances almost three times equivalent to the intended scene length. Where 14.97 meters was found to be the largest distance between the skeletal elements at scene one, the sternal end of the 6th right rib was found to exhibit the smallest at 8.76 meters. Therefore, this scene exhibited a range of 6.21 meters: the largest of the three scenes.

Comparatively, the average distance between both sets of skeletal elements at scene two was approximately 29.52 meters, with the right 10th rib showing the smallest distance between each feature at 28.18 meters and the left proximal clavicle demonstrating the largest at 30.04 meters. Such a range (28.18–30.04 meters) further depicts that the spatial relationships within this scene had the least variability in comparison to the others. This variance of 1.86 meters was closely followed by scene three, which exhibited a range of 47.63–49.52 meters (a 1.89 meter difference). However, the spatial relationships at this scene could not be ascertained as confidently as those of the open field terrains. With the dGPS obtaining only four datapoints from the skeletal elements, and the total station only plotting four variations of coordinates, spatial relationship analysis in ArcGIS Pro could only be conducted using four datapoints. Despite having the second smallest range, scene three exhibited the greatest distance between each set of skeletal elements. It was determined that the features at such terrain had an average distance of 48.11 meters, with the skull being the furthest at 49.52 meters and the left distal femur being the closest at 47.63 meters.

## 4 DISCUSSION

The application of a multidisciplinary approach at a forensic scene is becoming standard, with publications continually praising its benefits [11, 12, 30]. The need to provide undeniable evidence is of paramount importance at scatter scenes where existing protocols are vague. Currently, the application of techniques at such outdoor scenes is in its formative stage and so provides opportunities to be defined and modified.

At any forensic scene, sketch plans are often conducted as an initial attempt at evidence preservation, as highlighted by Dupras et al. (2012) [31]. With both methods depicting complementary skills, this study determined that a plotted scene sketch alongside the trilateration method is essential at a scatter scene. The use of two methods allows for the scene to be documented more intricately, minimizing human error and further indicating that without the use of either technique, scene reconstruction would yield lower accuracy.

Moreover, the concept of applying terrestrial surveying techniques to a forensic scene is not new and is frequently studied to ascertain their usefulness in different environments [32, 33, 34, 35]. Findings regarding the use of a differential GPS mostly corresponded to Walter and Schultz (2013) [33], who recommended this equipment only be used to map skeletal elements in open field environments. While the authors reasoned that this was due to a lack of research pertaining to obstructed terrains, this study expanded on such topics and established that here, a differential dGPS was highly incompatible, concluding that its use should only be for open field environments (if at all used).

Knowledge regarding prior uncertainties with the use of terrestrial surveying techniques, particularly with dGPS, led to the remains being positioned in resemblance to anatomical position, making it easier to determine any anomalies/discrepancies within the spatial analysis of these techniques and further clarify the extent to which they were observed. The degree of accuracy obtained by the dGPS was also able to be observed. Being 1 meter, the error level reduced confidence within the findings, reiterating the impracticability of this technique at scatter scenes where precise readings are required. The high error level can be explained through factors including satellite geometry and the presence of dense woodland and, therefore, subsequently provides an explanation into the inadequate data collection at wooded terrain.

Similarly, the total station findings were found to support the literature published by Berezowski et al. (2018) [36] and Raneri (2018) [37], who argue that at forensic scenes, prism offset total stations should be replaced with those that are reflectorless. Where Berezowski et al. (2018) and Raneri (2018) found the reflector to diminish the integrity of the scene, this study found that this tool could be used in a manner that aided scene preservation. Instead, the reflector only proved problematic due to the considerable amount of care required to ensure it was *exactly* perpendicular to the feature being recorded, as when held at even a slight angle, the likelihood of incorrect data being obtained increased dramatically. Having an error level of 0.002 m, the accuracy buffer was negligible on ArcGIS Pro and therefore provided no indication that data was plotted incorrectly at any scene. Although the error level of the total station is minute in comparison to the dGPS, the use of such instrumentation is impacted by fewer factors (such as the weather and range of the instrumentation [28]), consequently increasing uncertainty and skepticism among the conducted analysis.

Flatter and open environments pose fewer challenges to the total station, while wooded terrain hinders its use substantially and greatly alters the capabilities of this instrumentation [38].

By mapping, visualizing, and analyzing the data in ArcGIS Pro, this study was mostly successful in achieving its aims and objectives. To address the first and second aim: the efficiency of ArcGIS Pro does not change where the terrain differs. Irrespective of the terrain, ArcGIS Pro allowed for almost immediate analysis and took less than a minute to import and display the necessary data. The data visible on the map was easily interchangeable between terrains, making the interpretation of each scene straightforward. While a combined total of 597 datapoints were acquired for analysis, ArcGIS Pro did not lag, buffer, or crash. Although the lack of data at scene three did hinder the analysis conducted, the efficiency of ArcGIS Pro still did not diminish. Here, the software simply could not be used to the same extent, through fault of instrumentation and their capabilities at wooded terrain. Even at this scene, ArcGIS Pro worked to its full ability to produce sufficient results that did not exhibit ambiguity or inconclusiveness. Therefore, this GIS software yields exceptional applicability and accuracy when used to document scatter scenes.

Furthermore, to address the third aim, it can be concluded that ArcGIS Pro is an exceptionally versatile and useful tool in the documentation of scatter scenes. Able to be utilized in both a qualitative and quantitative manner, it can be employed in conjunction with additional scene documentation techniques to support and solidify their findings [39]. Where traditional mapping techniques provide an insight into the positioning of the skeletal elements and immediate scene vicinity, the visualization of datapoints within ArcGIS Pro provides greater context into the surrounding environments of the scene. Where accuracy buffers provided some evidence into the reliability of the terrestrial surveying technique used, spatial analysis proved to be easily adaptable, suggesting it could be applied to sites where skeletal elements have been distributed over a larger area. Not only could this technique aid in establishing exactly how far the skeletal elements have been distributed, but it may also help in determining why or how they came to rest in such a location, based on their distance from factors such as public paths, civilization, and ingress and egress routes. However, the use of ArcGIS Pro was found to be dependent on terrestrial surveying techniques, and so it cannot be excluded that the quantitative use of GIS can be improved given these are not utilized. Often a weighted/calculated influence of various GIS‐based entities can be used to identify a likelihood that corresponds to the research question. Influenced by Ruffell and Mcallister (2015) [40], Somma et al. (2018) [25] used GIS‐based entities such as vegetation, visibility, and landscape slope to search for clandestine graves in Italy and assessed their influence on the probability of such features being present.

In addressing the aims and objectives, it is evident that the integration of traditional archaeological mapping techniques and terrestrial surveying methods enabled GIS to be recognized as an appropriate tool in the documentation of scatter scenes. Limitations such as the lack of literature on surveying techniques at scatter scenes made it difficult to predetermine many of the technical difficulties or pitfalls experienced. Therefore, any issues that were encountered took longer to resolve, adding to the time it took to complete each scene. Furthermore, the absence of satellite signals within the wooded terrain proved to be detrimental to the dGPS findings, preventing complete analysis from being conducted. Lastly, this study solely focused on two terrains—the open field and woodland. While this was an appropriate representation of sites where scatter scenes are most prevalent, it is not inclusive of all scenes where scattered human remains can be found and risks decreasing the repeatability of this study.

## 5 CONCLUSION

### 5.1 Summary

Scatter scenes themselves have little representation both within the field of archaeology and among scholars. With current studies providing minimal structure regarding the documentation protocols at these scenes, this study was designed with the intention of closing the research gap. Through an integrated assessment, with a focus on GIS, the methods best suited for the documentation of scatter scenes were established. This research revealed that GIS serves as a useful tool in the documentation of scatter scenes, enabling investigators to adopt a qualitative or quantitative approach as required. With sufficient expertise on such software, correct data input and coordinate conversions, the placement of skeletal elements is easy to display, analyze, and interpret. Moreover, the terrain environment is not critical to the usefulness, accuracy, or efficiency of GIS, as this is instead affected by external factors. Furthermore, a multidisciplinary approach is crucial in the courtroom, and so the application of GIS to a scatter scene should not supplant current documentation methods. Alternatively, the software should be considered as an efficacious addition to such methods, optimizing the efforts of the forensic archaeologists.

### 5.2 Future recommendations

To expand and advance this study, future recommendations can be proposed. This study was conducted using techniques that could be accessed through Cranfield University. Employing additional techniques such as laser scanning, drones, and a reflectorless total station is recommended to establish the suitability of other surveying techniques at scatter scenes. Additionally, the repeatability of this study can continue to be assessed through applying this protocol to differing terrain and locations. However, it is recommended that these factors remain specific to scatter scenes, as areas such as the beach and desert would not be as favorable in comparison to mountainous terrain, forests, and hedge clearings. Furthermore, to best determine how GIS can be optimized at scatter scenes, further research into the alternative uses of this software should be conducted. It is recommended for such research to be tailored toward the tools available for quantitative analysis, such as probability mapping, though studies are not limited to this. Lastly, the addition of clothing and other items such as plastic bags containing skeletal elements  could be studied to determine their impact on the instrumentation when recording the position of skeletal elements.

## CONFLICT OF INTEREST STATEMENT

The authors have no conflicts of interest to declare.

## Data Availability

The data that support the findings of this study are available from the corresponding author upon reasonable request.
